# The Paris Dental Hospital

**Published:** 1915-08

**Authors:** D. O. M. Le Cron


### THE PARIS DENTAL HOSPITAL.

A communication comes from Dr. D. O. M. Le Cron, formerly of St. Louis, but now of the American Hospital in Paris, in which is described briefly conditions existing there and the experience he is having in the treatment of fractures and dislocations.

You will note by the above heading that I am connected with this hospital, doing work for humanity and giving my little mite toward relieving the poor suffering French soldiers.

We put in long hours from 8 A. M. to 6 P. M. seven days in the week—not much play, I assure you. We are all giving our service free and pay our own expenses. I feel I am contributing my pro rata in service rendered if not in cold cash.

They are trying to get Robert to come over and take my place as I can't afford to help them any longer; and they say it is difficult to get men that can conceive ideas for making appliances for fractured and horribly shattered jaws, and to construct them.

Dr. W. C. Roberts of London and I work together and we have had since here 29 cases of fractured and torn out jaws; in two cases the whole chin was torn away. It is remarkable what restorations we can make, and, let me say, the general surgeons here surely appreciate our work and give us due credit for same. This dental department that was started by Dr. Hayes and Dr. Wm. Davenport, of this city, will surely make much new history for dentistry and certainly make the M. D.'s step down and note that dentistry is an honorable and great profession.

Dr. Hayes gives his whole time and Dr. Davenport seven half-days a week. They have given their time since last September and will continue the good work 'till the completion of the war.

They started with one chair—we have six chairs and are now prepared to put in four to six more to meet the demands of the wounded that are coming in every day. We not only receive them from our hospital but ten other hospitals from this city have made application to send jaw cases to our department.

That alone is an open testimonial that our work in the dental department is becoming quite well known and the surgeons of the French hospitals are recognizing our department for its good work.

I have written Dr. Don Gallie and Dr. Burkhardt and made an appeal to the profession of America to contribute their mite in money to help our department. We get a small pro rata of the general fund but so little that we are all obliged to go down in our pockets, besides giving our service and expense, free to meet the demand.

It is too much to ask from Dr. Hayes, Dr. Davenport and others who are giving their service, also to contribute money to run the department. It takes quite a lot of money and they are obliged to depend on contributions from some source. All money from dentists should be to the Dental Department. If sent to the American Red Cross Society but little good will it do for the Dental Profession.

Here you see the real horrors of this great war and you on that side of the pond can't realize the destruction.

This city, once termed "Gay Parrie," now can aptly be termed the City of Mourning. Poor bleeding France with its many mutilated young men, sad-faced women in mourning and fatherless children, makes one's heart sick.

Today France has over three million of her young men at the front and over two million more in the field drilling and preparing to go to the front at their country's call to fill the vacant places of veterans that are slaughtered in this hellish war.—D. O. M. Le Cron, in *International Journal of Orthodontia.*

